# Enhancement of L-3-hydroxybutyryl-CoA dehydrogenase activity and circulating ketone body levels by pantethine. Relevance to dopaminergic injury

**DOI:** 10.1186/1471-2202-11-51

**Published:** 2010-04-23

**Authors:** Emilie Cornille, Mhamad Abou-Hamdan, Michel Khrestchatisky, André Nieoullon, Max de Reggi, Bouchra Gharib

**Affiliations:** 1 Neurobiologie des Interactions Cellulaires et Neurophysiopathologie, UMR CNRS 6184, Université de la Méditerranée, 13015 Marseille, France; 2 Institut de Biologie du Développement, UMR CNRS 6216, Université de la Méditerranée, 13009 Marseille, France

## Abstract

**Background:**

The administration of the ketone bodies hydroxybutyrate and acetoacetate is known to exert a protective effect against metabolic disorders associated with cerebral pathologies. This suggests that the enhancement of their endogenous production might be a rational therapeutic approach. Ketone bodies are generated by fatty acid beta-oxidation, a process involving a mitochondrial oxido-reductase superfamily, with fatty acid-CoA thioesters as substrates. In this report, emphasis is on the penultimate step of the process, i.e. L-3-hydroxybutyryl-CoA dehydrogenase activity. We determined changes in enzyme activity and in circulating ketone body levels in the MPTP mouse model of Parkinson's disease. Since the active moiety of CoA is pantetheine, mice were treated with pantethine, its naturally-occurring form. Pantethine has the advantage of being known as an anti-inflammatory and hypolipidemic agent with very few side effects.

**Results:**

We found that dehydrogenase activity and circulating ketone body levels were drastically reduced by the neurotoxin MPTP, whereas treatment with pantethine overcame these adverse effects. Pantethine prevented dopaminergic neuron loss and motility disorders. In vivo and in vitro experiments showed that the protection was associated with enhancement of glutathione (GSH) production as well as restoration of respiratory chain complex I activity and mitochondrial ATP levels. Remarkably, pantethine treatment boosted the circulating ketone body levels in MPTP-intoxicated mice, but not in normal animals.

**Conclusions:**

These finding demonstrate the feasibility of the enhancement of endogenous ketone body production and provide a promising therapeutic approach to Parkinson's disease as well as, conceivably, to other neurodegenerative disorders.

## Background

A frequent feature of neurodegenerative diseases is the impairment of glucose metabolism, the primary post-embryonal cerebral energy source (review in [1]). However, the brain also has the ability to adapt its metabolism and increase its reliance on lipids for energy production, through the fatty acid *β*-oxidation pathway. The process involves L-3-hydroxyacyl-CoA dehydrogenase (HAD) (EC 1.1.1.35) activity which generates ketone bodies (KBs) [2,3]. The end product, acetyl-CoA, feeds into the TCA cycle to produce energy in the form of NADH and FADH_2_. The KBs *β*-hydroxybutyrate (*β*-OHB) and acetoacetate (ACA) have a protective role in a broad spectrum of cerebral injuries and diseases and they preserve neuronal cell integrity and stability in vitro [4-6]. The experimental approaches used are intravenous infusion of mice or rats with *β*-OHB [1,7] or administration of a ketogenic diet. The utilization of *β*-OHB by the brain and other tissues is contingent on its dehydrogenation to ACA. Little is known, however, about what events in diseased animals, regarding changes in *β*-oxidation rate and KB levels associated with cerebral disorders.

We therefore investigated changes in fatty acid *β*-oxidation in the MPTP mouse model of Parkinson's disease (PD). The disorders result from a multifactorial cascade of deleterious factors [8] leading to a progressive degeneration of dopaminergic neurons of the substantia nigra and resulting in motor troubles. A hallmark of the pathological process is the decrease of mitochondrial function, in particular the inhibition of electron transport system complex I [9-12] associated with oxidative stress. Depletion of GSH levels, observed in the substantia nigra of PD patients, as well as in the MPTP model, is one of the earliest events leading to the inhibition of complex I and subsequent mitochondrial dysfunction [13,14].

Experimental data published already in the 1980's indicated that pantethine, a low-molecular-weight thiol widely distributed in the living world, circumvents the impairment of fatty acid *β*-oxidation in rat liver mitochondria and microvessels of the brain [15,16]. Pantethine is also well known to stimulate lipolysis and, conversely, to inhibit fatty acid synthesis [17]. MPTP-intoxicated mice display impaired energy production [18], that is improved by KB infusion [19]. We therefore investigated the effects of pantethine treatment in this model. We determined the changes in the NAD-dependent conversion of 3-hydroxybutyryl-CoA to acetoacetyl-CoA. Our main finding is that i) the dehydrogenase activity and circulating KB levels decreased in intoxicated mice and ii) these parameters were restored by pantethine treatment, with improvement of dopaminergic injury and functional disorders.

## Methods

### Reagents

Unless otherwise mentioned, all chemicals and reagents were obtained from Sigma-Aldrich Chemical Company (St. Louis, MO) as the highest available grades.

### Animals and treatment

Ten-week old male C57BL/6 mice were purchased from Janvier (Le Genest, France). They were handled according to the rules of "Décret #87-848 du 19/10/1987, Paris"; approval #007031. Mice were administered 1-methyl-4-(2'-methylphenyl)-1,2,3,6- tetrahydropyridine (abbreviated as 2'-methyl-MPTP), a more potent neurotoxin in mice than MPTP [20,21]. The neurotoxin was given via i.p. injections, at 20 mg/kg, according to a method described previously [22]. Unless otherwise mentioned, two 2'-methyl-MPTP injections were performed on the same day at a 6 h interval, followed by one injection on the following day. This protocol caused an about 80% loss of striatal dopamine levels. D-pantethine was indicated by the manufacturer to be 97% pure. Purity of the preparation was further ascertained using HPLC-mass spectrometry analysis (LCUV-MSD analysis) (SCA CNRS, Lyon, France). Mice received two 5-day treatments with daily i.p. injections of 15 mg of pantethine, before and after 2'-methyl-MPTP administration. Preliminary experiments showed that this regimen was well tolerated and gave the best results for an as short as possible treatment. Intraperitoneal administration avoids extensive hydrolysis of pantethine by intestinal pantetheinase. The high dosage used is made necessary by the quick elimination of pantethine into the urine [23]. When used, pantothenate was injected at a molar concentration equivalent to that of pantethine; for cystamine, the highest non-toxic dose was used, i.e. 1.875 mg per mouse. The solutions were prepared in apyrogenic 0.9% NaCl solution. Control animals were injected with the same saline solution.

Pantethine reduction to pantetheine was performed using the disulfide reducing gel TCEP (Tris (2-carboxyethyl) phosphine) according to the manufacturer's instructions (Pierce Chemical, Rockford, IL). Briefly, the gel was washed with 50 mM pH 7.5 phosphate buffer and then incubated with pantethine solution for 1 h at room temperature. Pantethine reduction was checked by scanning the UV-absorption spectrum using a Beckman DU800 spectrophotometer. The decrease of the 245 nm-peak until complete extinction indicated complete reduction.

### Isolation of mitochondria

Brain and liver tissue were homogenized in mitochondria extraction buffer (IMGENEX, Cliniscience, Montrouge, France) according to the manufacturer's instructions. Brain mitochondria were isolated using a discontinuous Percoll gradient [24]. After a 10 min centrifugation at 30 400 × g, the mitochondrial layer was collected and washed 3 times with 10 ml of extraction buffer. Liver mitochondria were prepared as described previously [25]. Mitochondrial pellets were then resuspended in a hypotonic buffer composed of (25 mM KH_2_PO_4 _and 5 mM MgCl_2_, pH 7.5). Protein concentration was determined by the Biorad assay method. Isolated mitochondria were checked for membrane integrity by assaying citrate synthase activity.

### Determination of L-3-hydroxybutyryl-CoA dehydrogenase activity and of circulating KB levels

We performed time course studies to assess 2'-methyl-MPTP-induced changes in fatty acid β-oxdation. L-3-hydroxybutyryl-CoA dehydrogenase activity was assayed both in the brain and liver, since the neurotoxin-induced injuries are not limited to the brain [26,27]. Control and pantethine-treated mice (15 mg/5 days) received two 2'-methyl-MPTP injections on the same day at 6 h intervals. Under these conditions, the neurotoxin induces an active neurological phase ranging from 12 hours to 4 days after the last injection, followed by a progressive recovery [28]. Brain and liver mitochondrial samples were prepared 1, 2, 4 and 7 days following MPTP injections. Dehydrogenase activity was determined in the forward direction, as it occurs in vivo [29]. We used 125 μM β-hydroxybutyryl-CoA as a substrate [30] in an assay medium containing 75 mM Tris (pH 10) with 75 mM KCl, NAD^+ ^1.2 mM. Reduction of NAD was followed at 340 nm, with an extinction coefficient taken as 6.22 mM^-1^cm^-1^. Control assays were conducted in the absence of the substrate. For determination of hydroxybutyrate and acetoacetate levels, blood samples were taken from the retro-orbital plexus of halothane-anesthetized mice into capillary tubes containing 3.8% sodium citrate. Blood was immediately deproteinized with 4% perchloric acid, centrifuged at 17 500 × g, 15 min at 4°C. Ketone bodies were quantified using enzymatic assay kits (Biosentec, Toulouse, France) based on the reduction of NAD or oxidation of NADH, followed at 340 nm in a spectrophotometer. The assays were repeated three times.

### Mass spectrometry analysis

Changes in brain GSH levels following pantethine injection were determined using HPLC-mass spectrometry analysis (LCUV-MSD analysis) (SCA CNRS, Lyon, France). Healthy mice received a single i.p. injection of 15 mg of pantethine and were then sacrificed at different times, from 30 min to 4 h. Blood samples were taken from the retro-orbital plexus of halothane-anesthetized mice into capillary tubes containing 3.8% trisodium citrate. Mice were then perfused with heparinized saline. Perfused brain and plasma samples were immediately homogenized in 3 volumes of 4% perchloric acid. Samples were centrifuged at 20,000 × *g *for 10 min at 4°C; the supernatants were snap-frozen in liquid nitrogen and stored at -80°C pending assay. Samples were analyzed using the HP1100-MSD (B) system with HPChemstation software, version A.08.01-682. Chromatographic separations were performed by reverse-phase chromatography on a 2 mm (inner diameter) C18 Waters Hilic column. A gradient of 0.05% formic acid pH 2.6/acetonitrile was delivered over 15 min to elute the compounds at a flow rate of 300 μl/min. Compounds were ionized by electrospray (ES+) ionization. The mass spectral data were processed into peak lists containing the characteristic [M+H]^+ ^ion m/z = 308.

### Brain glutathione assays

Saline or pantethine-treated mice were anesthetized with halothane on days 1 and 7 following the last neurotoxin injection. Brain samples were frozen immediately in liquid nitrogen and homogenized in 9 volumes of 100 mM potassium phosphate buffer pH 7.5 containing 1 mM EDTA. An aliquot of the homogenate was added to an equal volume of 5-sulfosalicylic acid (1% w/v). After centrifugation at 8,000 *g *for 10 min, the total glutathione (GSH + GSSG) concentration in the supernatant was determined with the colorimetric GSH assay Kit (ApoGSH Glutathione Colorimetric Assay Kit, MBL, Woburn, MA) according to the manufacturer's instruction.

### Mitochondrial function

#### Complex I activity

For in vivo treatment, brain mitochondria were isolated from mice treated with 15 mg of pantethine for 5 days. For in vitro treatment, pantethine or pantetheine were added to mitochondria isolated from control mice. Complex I activity was determined based on protocols described previously [31]. Mitochondria were lysed by freeze-thawing three times. To initiate the reaction, 150 μg-protein extracts were added to the hypotonic assay buffer containing (65 μM ubiquinone_1_, 130 μM NADH, 2 μg/ml of antimycin A, and 2.5 mg/ml of defatted BSA), in the presence of different concentrations of MPP^+ ^(0, 1.25, 2.5 mM). Pantetheine or pantethine (0, 0.5 or 1 mM) was added 5 min later. The oxidation of NADH was monitored spectrophotometrically at 340 nm for 3 min at 30°C prior to the addition of the complex I inhibitor rotenone (2 μg/ml), after which the activity was measured for an additional 3 min. The differential rate before and after the addition of rotenone was used to calculate complex I activity. We checked, using the Ellman's reagent, that the reduced form pantetheine was maintained throughout the experiment.

#### Polarography

Brain mitochondria were suspended in respiration buffer consisting of (225 mM mannitol, 75 mM sucrose, 10 mM KCl, 5 mM HEPES, 5 mM K_2_HPO_4 _pH 7.5), with freshly added 1 mg/ml of defatted BSA at 30°C. Oxygen consumption was measured in a closed-chamber cuvette with a mini-stirring bar using a Clark-type electrode (VWR Fontenay sous Bois, France). To assess complex I-mediated mitochondrial respiration, a one mg-protein preparation in 1 ml of respiration buffer was preincubated with 10 mM glutamate and 5 mM malate in the presence of MPP^+ ^(0, 50 and 100 μM) at 30°C. After 5 min of incubation, pantetheine at (0, 0.5 or 1 mM) was added and the oxygen consumption was measured for an additional time before the addition of 500 uM ADP to induce state 3 respiration. Oxygen consumption was monitored throughout the experiment.

#### ATP measurements

Samples were prepared under conditions identical to those of the polarographical study. Mitochondria suspended in respiration buffer were incubated in the presence of MPP^+ ^(0, 50 or 100 μM). Pantetheine (0, 0.5 or 1 mM) was added 5 minutes later. After the reaction was stopped, the mitochondrial suspension was lysed in an equal volume of lysis buffer from the ATP bioluminescence assay kit (Sigma), and the content of ATP was measured according to the manufacturer's instructions. Light emitted from luciferase-mediated reaction was captured in a Beckman DTX800 luminometer and calculated from a log-log plot of a standard curve of known ATP concentrations.

### Striatal dopamine levels and motor activity

Saline and pantethine-treated mice were sacrificed by decapitation on day 7 after the last 2'-methyl-MPTP administration and the striata were collected and stored at -80°C until analysis. The endogenous levels of dopamine (DA) and its metabolites, dihydroxyphenyl acetic acid (DOPAC) and homovanillic acid (HVA), were determined using high performance reverse phase liquid chromatography with electrochemical detection according to the method described previously [32]. Before being sacrificed, the mice were used for the determination of hypokinesia-like symptoms, using the iron pole test according to the method described previously [33,34]. After pre-trial acclimatization, each mouse was given six consecutive trials and the mean of the three best trials was retained. Animals which died before the test were noted as having required 60 sec.

### Tyrosine hydroxylase (TH) immunohistochemistry and quantitative analysis

Mice received two 2'-methyl-MPTP injections on the same day at 6 h interval, followed by one injection on the following day. They were treated with saline or pantethine for 5 days, before and after the neurotoxin injections, and they were then sacrificed by decapitation. Brain coronal sections (10 μm), cut every 100 μm, were incubated with a rabbit anti-TH antibody (Calbiochem-Novabiochem, San Diego, CA) (1:1000) followed by peroxidase-conjugated anti-IgG antibody (Jackson ImmunoResearch, obtained from Beckman Coulter, Marseille, France). Immunoreactivity was visualized by incubation in 0.1% 3,3'-diaminobenzidine tetrahydrochloride (DAB) and quantified using LUCIA image analysis software (Laboratory Imaging, Prague, Czech Republic). For the determination of dopaminergic cell loss, the sections were counterstained with cresyl violet (Sigma-Aldrich). The number of TH-positive neurons in the substantia nigra pars compacta (SNpc) was estimated using a semi-quantitative method [35]. The area of the SNpc was determined at low magnification (4× objective) and neurons were counted at higher magnification (40× objective) in three microscopic fields (area of 40,000 μm2 each) situated on the mediolateral axis of the SN of each histological section. Neuronal density was calculated as the ratio between the sum of neuronal counts and the sum of the areas of 5 sections and is expressed as number of neurons per square millimeter.

### Statistical analysis

Nonparametric Mann-Whitney U tests were performed using GraphPad Prism software. Dopamine levels and motor activity time data were analyzed using the Newman-Keuls test for groups; *p *< 0.05 was considered significant.

## Results

### L-3-hydroxybutyryl-CoA dehydrogenase activity and circulating KB levels

**Figure 1:**
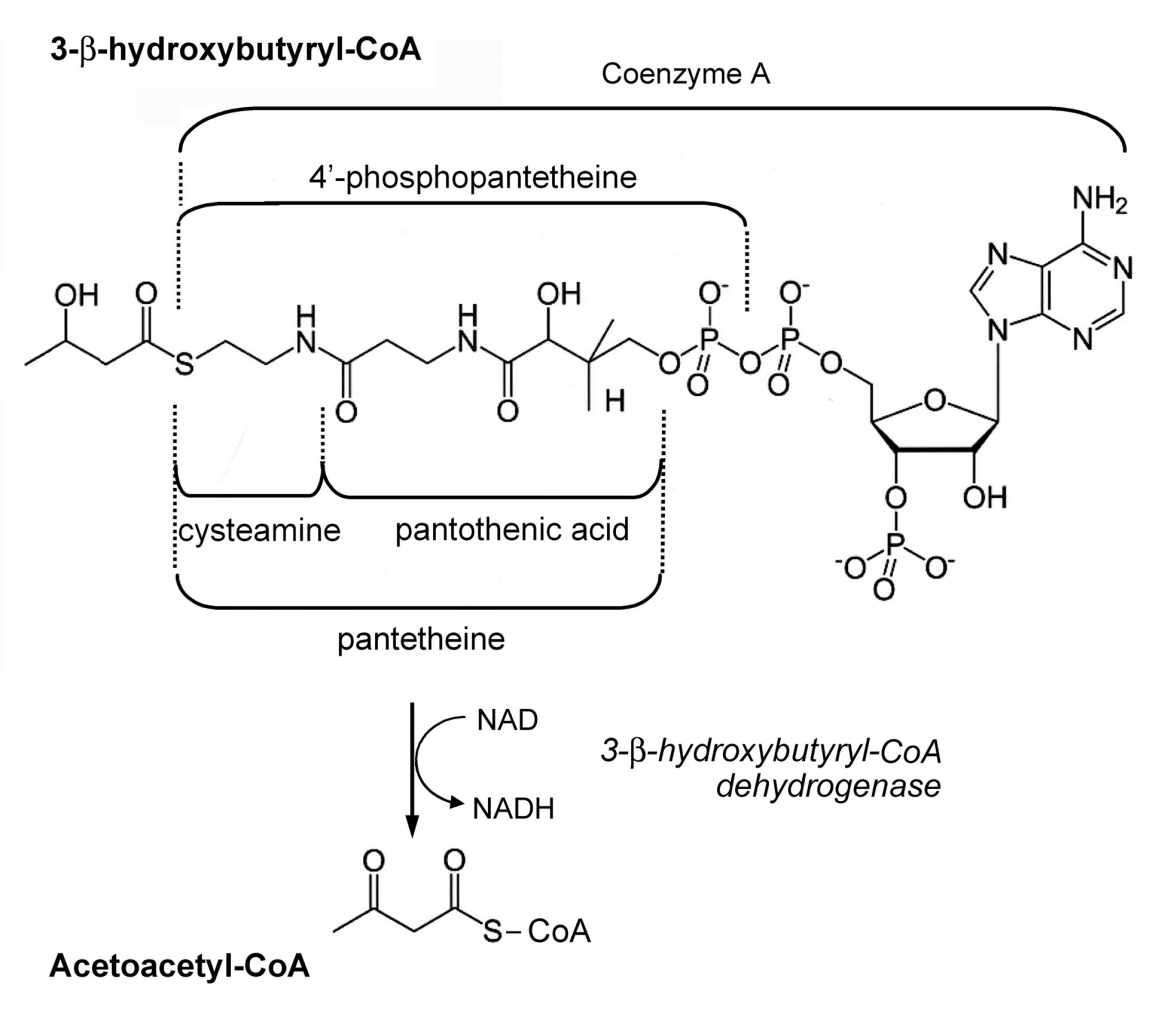
**Dehydrogenation of 3-hydroxybutyryl-CoA to acetoacetyl-CoA**.

**Figure 2:**
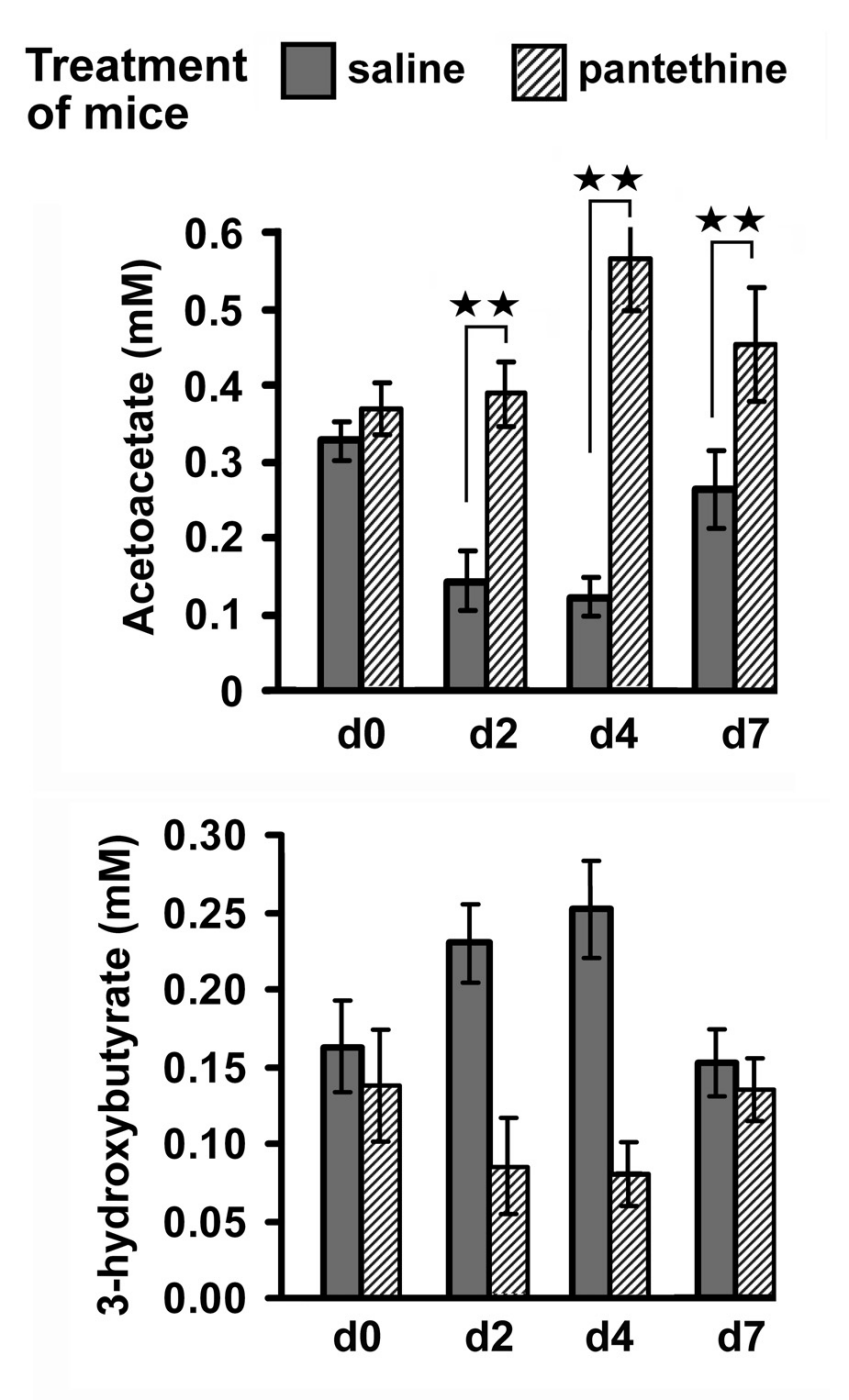
**Effects of pantethine treatment on circulating ketone body levels**. Changes in circulating levels of KBs (3-hydroxybutyrate and acetoacetate) in 2'-methyl-MPTP-intoxicated mice, treated with pantethine or saline. d2-d7, days following injection of the neurotoxin; n = 4 mice per assay. Significant difference between pantethine-treated versus saline groups, **p < 0.01.

Time course studies were performed to assess 2'-methyl-MPTP-induced changes in L-3-hydroxybutyryl-CoA dehydrogenase activity in brain and liver mitochondria. The animals were treated with either pantethine or saline. In the forward direction, yielding NADH and acetoacetate (ACA), enzymatic activity was determined using L-3-hydroxybutyryl-CoA as substrate (fig 1). In the brain, the activity was reduced drastically on day 1 following 2'-methyl-MPTP injection (table 1). In the liver, the enzyme activity was about two orders of magnitude higher than in the brain and it was also inhibited by the neurotoxin, with however a different time course: minimal activity was observed on day 4. Pantethine treatment mitigated the inhibitory effect of the neurotoxin, and always maintained enzymatic activity close to the control level in both brain and liver. Under the mild pathological conditions used in this study, activity tended progressively back to normal levels on day 7. Changes in circulating KB levels were consistent with the changes in enzyme activity in the liver, responsible for the circulating KBs. In agreement, the concentration of ACA, the enzyme product, was drastically reduced when dehydrogenase activity was inhibited, i.e. on days 2-4 following the neurotoxin injection (Fig. 2). In contrast, the fall of ACA concentration was avoided in pantethine-treated animals; it even displayed a drastic rise on day 4, reaching a level about four times higher than in paired saline controls. The concentration of the enzyme substrate, *β*-OHB, correlated negatively with the ACA concentration. Pantethine had little effect on dehydrogenase activity and KB levels in healthy animals. Under our experimental conditions, mice treated with pantothenic acid or cystamine instead of pantethine did not differ from the saline groups (data not shown).

**Table 1:** Effects of pantethine treatment on L-3-hydroxybutyryl-CoA dehydrogenase activity.

Days after 2'methyl -MPTP injection	L-3-hydroxybutyryl-CoA dehydrogenase activity			
	
	Brain (nmol.min^-1^.mg protein^-1^)		Liver (μmol.min^-1^.mg protein^-1^)	
	**Saline**	**Pantethine**	**Saline**	**Pantethine**
d0	63.91 ± 4.90	73.95 ± 2.55#	2.96 ± 0.35	3.11 ± 0.27
d1	28.91 ± 3.54**	51.09 ± 4.87##	2.74 ± 0.14	3.56 ± 0.18#
d2	35.58 ± 3.31**	45.82 ± 3.84#	2.08 ± 0.17**	2.94 ± 0.19##
d4	45.01 ± 5.19*	46.01 ± 5.05	1.60 ± 0.27**	2.98 ± 0.32##
d7	54.31 ± 4.95	57.55 ± 3.65	2.67 ± 0.18	2.85 ± 0.17

Brain and liver mitochondria were isolated from 2'-methyl-MPTP- injected mice, previously treated with saline or pantethine. Tissue and blood samples were collected from day 0 to day 7 following the neurotoxin injection and were then processed for dehydrogenase activity, i.e conversion of L-3-hydroxybutytyl-CoA to acetoacetyl-CoA. Values are means ± SD; n = 4 mice per assay. Significant decrease versus d0 control, *p < 0.05 and **p < 0.01; significant increase versus paired saline, #*p *< 0.05 and ##*p *< 0.01.

### GSH stores

**Figure 3:**
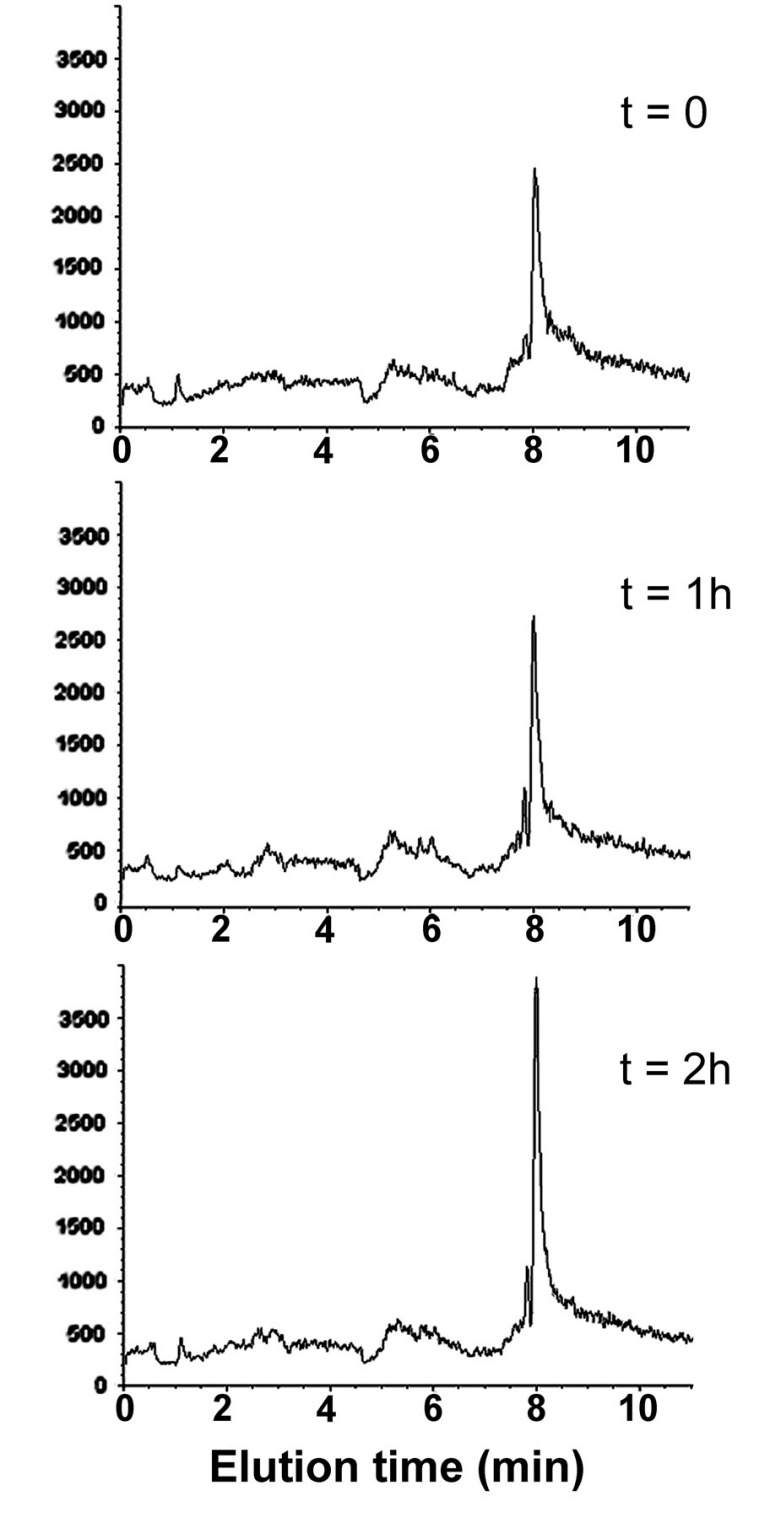
**Increase in brain GSH levels following pantethine injection**. Normal mice received a single i.p injection of 15 mg of pantethine and were sacrificed at different times. Brains were perfused and sampled for analysis by LCUV-MSD. The figure shows the mass spectral data processed into peak lists containing the characteristic [M+H]^+ ^ion m/z = 308 for gluthatione.

HPLC-mass spectrometry analysis showed that administration of pantethine rapidly increased GSH levels in normal animals: a single pantethine injection triggered a rise in brain GSH levels within 2 hours (Fig. 3). A similar GSH rise was also observed in plasma (not shown). After intoxication with 2'-methyl-MPTP, GSH stores in the brain were reduced by about 35%, whereas they remained at the control levels in pantethine treated ones (Table 2).

**Table 2:** Preservation of GSH stores by pantethine treatment.

	Days following 2'-methyl-MPTP injection	
	**Day 1**	**Day 7**

Control	2.00 ± 0.13	1.81 ± 0.09
2'-methyl-MPTP + saline	1.25 ± 0.18	1.66 ± 0.20
2'-methyl-MPTP + pantethine	1.96 ± 0.12^§^	1.79 ± 0.10
Control + pantethine	2.54 ± 0.29#	

Total brain glutathione levels were determined in 2'-methyl-MPTP- intoxicated mice, treated with either saline or pantethine. Glutathione levels are expressed as μmol/g of tissue. Values are means ± SD; n = 6 mice per assay. Significant increase versus paired saline, ^§ ^*p *< 0.05, and versus the control group, #*p *< 0.05.

### Mitochondrial activity

**Figure 4:**
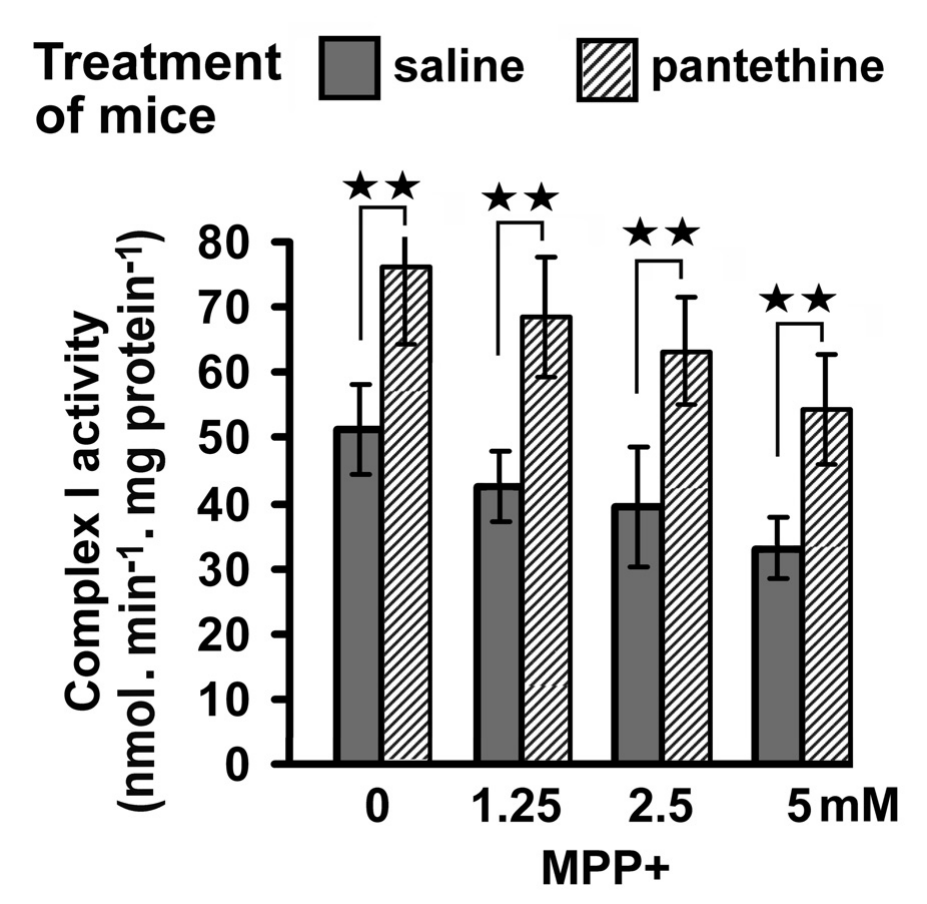
**Preservation of mitochondrial complex I activity by pantethine treatment**. Mitochondria were isolated from saline or pantethine-treated mice and incubated with 0, 1.25, 2.5 and 5 mM of MPP^+ ^for 5 min, then complex I activity was determined. Values are means ± SD; n = 4 mice per assay. Significant difference between pantethine-treated versus saline groups, **p < 0.01.

**Figure 5:**
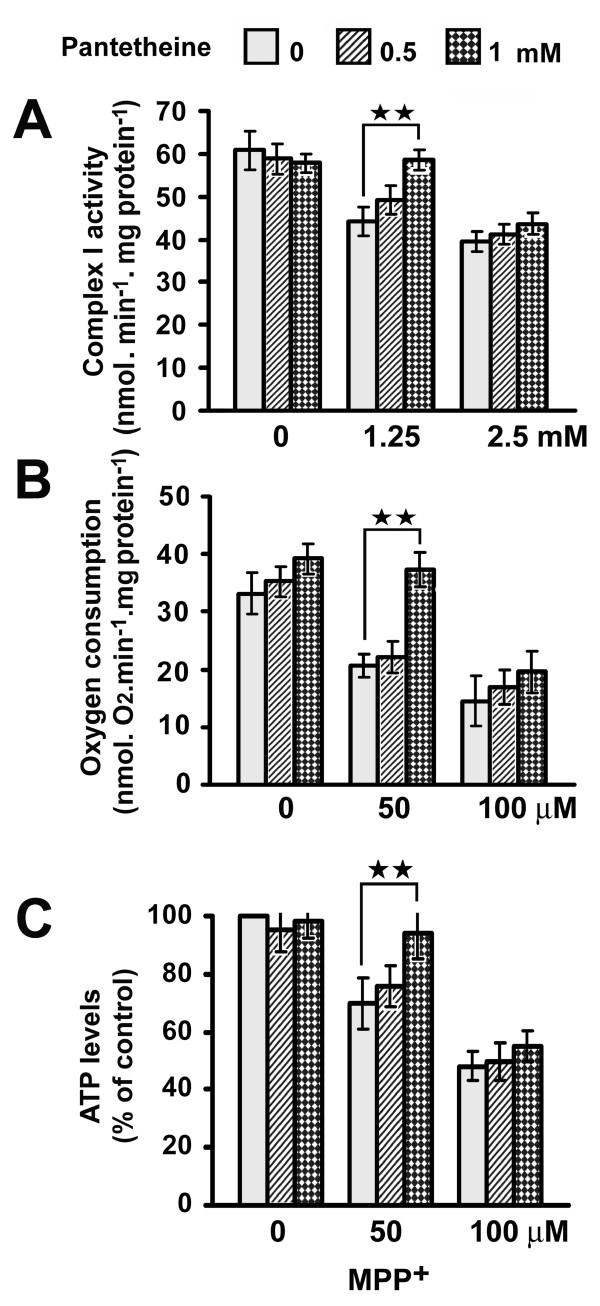
**In vitro improvement of mitochondrial functions by pantetheine**. Mitochondria were isolated from control mice and incubated with increasing concentrations of MPP^+ ^for 5 min; 0, 0.5 or 1 mM pantetheine was then added and **(A) **complex I activity, **(B) **oxygen consumption and **(C) **ATP levels were determined. Values are means ± SD; n = 4 mice per assay. Significant difference between assays in the presence of the absence of pantetheine, **p < 0.01.

NADH-ubiquinone oxidoreductase (respiratory chain complex I) activity was determined in brain mitochondria. In a first experiment, mitochondria isolated from mice treated with either pantethine or saline were incubated with MPP^+^, the active metabolite of MPTP. MPP^+ ^reduced complex I activity in a dose-dependent manner. The activity was however significantly enhanced by pantethine treatment: whatever the MPP^+ ^concentration used, the activity was always higher in mitochondria from pantethine-treated mice than in the saline matched pairs (Fig. 4). In a second experiment, we examined the effects of pantethine on mitochondria isolated from control mice, in the presence of MPP^+^. In this case, restoration of complex I activity was obtained not with pantethine but with its reduced form, i.e. pantetheine. At a 1 mM concentration, pantetheine re-established completely complex I activity in the presence of 1.25 mM MPP^+^. At a lower pantetheine concentration (0.5 mM) or in the presence of a higher MPP^+ ^concentration (2.5 mM) no protective effect was observed (Fig. 5A). We further examined whether pantetheine might rescue mitochondrial respiration and ATP production depressed by MPP^+^-mediated complex I blockade. Under our experimental conditions, glutamate- and malate-supported oxygen consumption was reduced by 38% in the presence of 50 μM MPP^+^; the reduction did not occur in the presence of 1 mM pantetheine (Fig. 5B). At a higher MPP^+ ^concentration (100 μM), where respiration was inhibited by 56%, pantetheine was ineffective. Pantetheine had similar effects on mitochondrial ATP levels: in the presence of 50 μM MPP^+^, it restored the normal ATP levels when used at a 1 mM concentration (Fig. 5C). In these in vitro experiments, the native form pantethine had no effect (data not shown).

### Dopaminergic structures and motor ability

**Figure 6:**
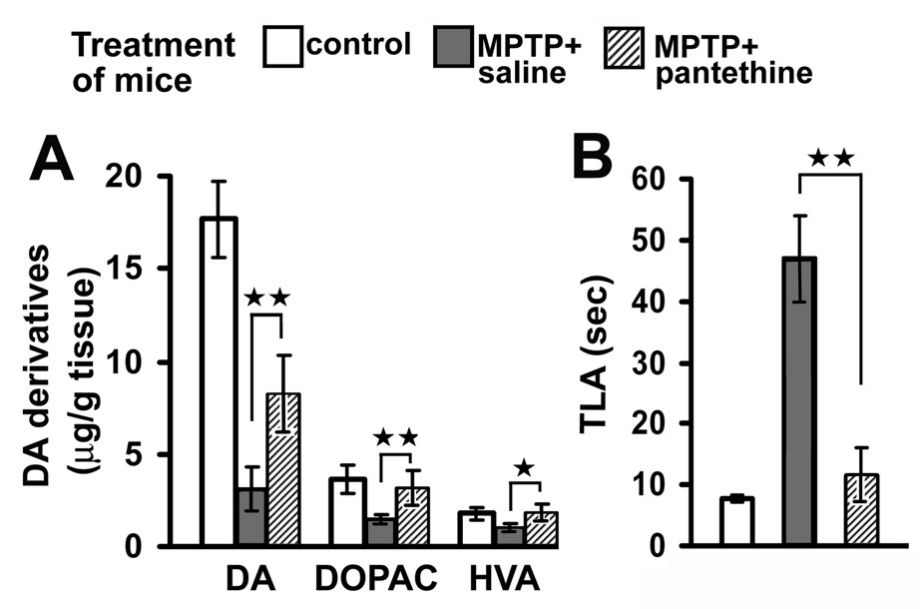
**Pantethine treatment preserves striatal dopamine levels and motor activity**. **(A)**, striatal levels of dopamine (DA) and its derivatives DOPAC and HVA. **(B)**, motor function evaluated in the pole test (TLA, motor activity time, indicates the time for the mice to go back to the ground). Values are means ± SEM, n = 10 mice per group. MPTP refers to 2'-methyl-MPTP. Significant difference between saline and pantethine-treated groups, *p < 0.05; **p < 0.01.

**Figure 7:**
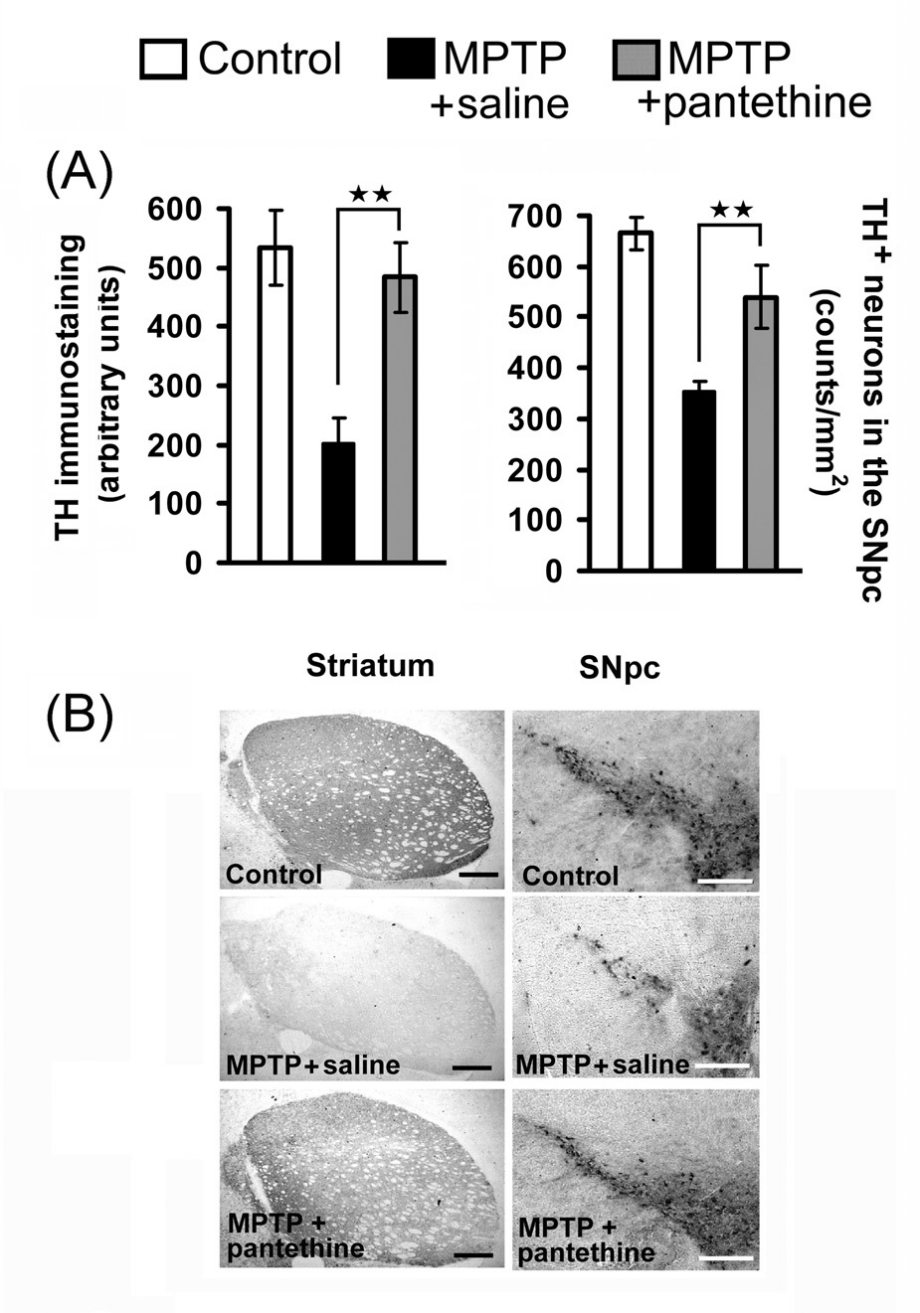
**Pantethine treatment rescues nigro-striatal dopaminergic injury**. **(A)**, histological analysis of TH^+ ^striatal fibers and TH^+ ^SNpc neurons in control and 2'-methyl-MPTP-injected mice, treated with pantethine or saline. Mice were sacrificed 7 days after the last injection of the neurotoxin. Pantethine treatment restored TH^+ ^striatal fibers and TH^+ ^SNpc neuron count to almost the control level. Error bars represent mean ± SD; n = 4 mice per group. Significant difference between the saline and pantethine-treated groups, **p < 0.01. **(B)**, representative TH immunohistochemical staining of striatum and SNpc in brain coronal sections. MPTP refers to 2'-methyl-MPTP. Scale bars: striatum, 400 μm; SNpc, 200 μm.

The overall effect of pantethine treatment was the reversal of 2'-methyl-MPTP-induced structural and functional defects. Mice were taken 7 days after neurotoxin injections and their motility was determined using the iron pole test. The animals were then sacrificed and the brains processed for quantification of dopamine (DA) and its metabolites. Under our experimental conditions, levels of striatal DA, DOPAC and HVA were significantly reduced, with DA levels being reduced by more than 80% (Fig. 6A). The motility of the animals in the iron pole test was dramatically impaired (Fig. 6B). In the pantethine treated group, the loss of striatal DA content was attenuated, to about 50% of the normal level. The treatment restored also the normal levels of DOPAC and HVA. Correlatively, the treated mice behaved like normal mice in the iron pole test. Healthy animals were not affected by the treatment (data not shown). The functional protection by pantethine against the toxicity of 2'-methyl-MPTP was associated with the preservation of the nigrostriatal structures. In the saline group, striatal tyrosine hydroxylase OD was reduced to 35% of the control level, whereas it was close to the values of controls in pantethine-treated mice (Fig. 7A). Accordingly, the number of SNpc TH+ neurons were significantly higher in pantethine-treated mice than in the saline group and were close to the values in the control group. Fig. 7B shows typical aspects of the brain structures involved.

## Discussion

We showed that the neurotoxin 2'-methyl-MPTP inhibited L-3-hydroxybutyryl-CoA dehydrogenase activity and caused a concomitant fall of circulating levels of the product of the reaction, acetoacetate (ACA). These alterations were avoided by pantethine treatment. The protective effect of pantethine was associated with the enhancement of GSH synthesis, restoration of mitochondrial function, i.e. complex I activity, ATP synthesis and oxygen consumption, leading to a protection against dopaminergic injury.

KBs are produced by hepatocytes and are transported to the tissues, including the brain; astrocytes are also ketogenic, although to a lesser extent however [36]. In agreement, we found that dehydrogenase activity is two-orders of magnitude higher in the liver than in the brain, and circulating KB levels correlate with enzyme activity of the liver. We observed that injection of the neurotoxin induced, in both brain and liver, a decrease of dehydrogenase activity, which was restored by pantethine treatment. These findings may be compared with published data on L-3-hydroxyacyl-CoA dehydrogenase type II/amyloid binding alcohol dehydrogenase (HADHII/ABAD). The enzyme is downregulated in PD patients and in the mouse, on days 2 to 7 after MPTP injection. Conversely, transgenic mice with increased expression of human HADHII/ABAD are significantly more resistant to MPTP; overexpression of the enzyme mitigates MPTP-induced impairment of oxidative phosphorylation and ATP production [37]. Thus the changes of dehydrogenase activity that we observed following MPTP injection and pantethine treatment may reflect changes in the amount of the enzyme.

Pantethine is metabolized in vivo, yielding mainly pantetheine, cysteamine, pantothenic acid and 4'-phosphopantetheine (4'-PP) [38]. All these derivatives may well be involved in the effects that we observed after treatment with pantethine. The treatment increased the GSH concentration, which is likely to be mediated by the increase of intracellular levels of L-cysteine via disulfide exchange reactions. Free cysteine is available to several pathways, including formation of mixed disulfides, and synthesis of glutathione [39]. The maintenance of normal GSH levels is essential for NADH-ubiquinone oxido-reductase (complex I) activity since the complex I is thiol-regulated [13,40,41]. Accordingly, we found that, when applied to isolated mitochondria, the reduced form pantetheine was able to preserve complex I activity, and therefore to maintain complex I redox status; the oxidized form pantethine did not. It should be underlined that GSH and CoA levels, as well as complex I activity and ATP production are interdependent and their interactions remain to be clarified [42,43].

Our main finding is that the administration of pantethine to 2'-methyl-MPTP intoxicated mice stimulates fatty acid β-oxidation and increases circulating KB levels.

This effect is specific for pantethine and was not observed with cystamine. This may reflect the fact that pantetheine constitutes the active moiety of CoA. Not only CoA-fatty acid thioesters but also pantetheine- and 4'PP-fatty acid thioesters are acceptable substrates for HAD. In comparison, cysteamine is a too small an entity; the HAD *K*m value is 125 fold higher for acyl-cysteamine than for acyl-pantetheine [44]. The thiol group and the pantoic acid moiety (2,4-dihydroxy-3-dimethyl butyric acid) of pantetheine play a central role in enzyme binding [44,45]. These elements do not occur in either pantothenic acid or cysteamine, respectively. Accordingly, under our experimental conditions, these two compounds were unable to enhance either dehydrogenase activity or circulating levels of KB (data not shown). Pantethine also displays anti-inflammatory activity in inhibiting the activation of the cellular response to pro-inflammatory factors, as we reported earlier [46].

In summary, treatment with pantethine reproduces the effects of KB administration and ketogenic diets with however several advantages. First, the stimulation of KB synthesis is a rational way to enhance KB levels. Second, long-term administration of high fat diets has detrimental effects [47] that could be circumvent by the hypolididemic properties of pantethine. Third, pantethine has apparently no effects on circulating ACA levels under normal conditions, meaning that it could act "on demand" only.

## Conclusions

It is now becoming clear that the cerebral hypometabolism in major diseases such as PD and Alzheimer's disease [48,49] constitutes a therapeutic target. Treatments able to enhance neuronal energy reserves may improve the ability of neurons to resist metabolic challenges [50] and may be effective in relieving physiological as well as cognitive dysfunctions. Pantethine seems to promote metabolic flexibility, i.e. the transition between carbohydrate and lipid utilization for energy production and is therefore a good candidate drug against diseases associated with metabolic disorders. Under our experimental conditions, we used high doses of pantethine; however, in view of a potential clinical application, an appropriate delivery device may improve the efficiency of treatment and allow a drastic reduction of the dose to be administered.

## Authors' contributions

EC, MAH: performed research. AN: involved in project conception and supervision; performed dopamine quantification. MK: involved in project conception and manuscript revision. MdR: involved in project conception, data analysis and interpretation, writing the paper. BG: involved in project conception, experiment design, data analysis and interpretation, writing the paper. All authors read and approved the final manuscript.
